# Response to Shen et al to rapid communication “Fact or fiction: Exploring resident mesenchymal stem cells in abdominal aortic aneurysm from multiple perspectives”

**DOI:** 10.1016/j.gendis.2025.101703

**Published:** 2025-06-03

**Authors:** Carmen Ciavarella, Francesco Alviano, Gianandrea Pasquinelli

**Affiliations:** Department of Medical and Surgical Sciences (DIMEC), Alma Mater Studiorum-University of Bologna, Bologna, Italy; Department of Biomedical and Neuromotor Sciences (DIBINEM), Alma Mater Studiorum-University of Bologna, Bologna, Italy; Department of Medical and Surgical Sciences (DIMEC), Alma Mater Studiorum-University of Bologna, Bologna, Italy; Pathology Unit, IRCCS Azienda Ospedaliero-Universitaria di Bologna, Bologna, Italy

We appreciate Shen and colleagues for their insightful communication regarding the presence and localization of mesenchymal stromal cells (MSCs) in the human abdominal aortic aneurysm (AAA) wall, and for citing our work.^1^ As they note, identifying MSCs in the human vascular wall is challenging due to the lack of universally accepted markers. This issue is particularly evident in injured tissues like AAAs, where inflammation, oxidative stress, hypoxia, and other stress factors coexist in the atherosclerotic environment.^2^ Moreover, vascular wall cell plasticity and differentiation processes contribute to the loss of cellular hierarchy, such as with endothelial-to-mesenchymal transition. This process induces endothelial cells to acquire mesenchymal stem cell characteristics with pro-calcific potential,^3^ further complicating MSC identification.

Our studies demonstrated the presence and functional dysregulation of MSCs in AAA tissues. Key findings include: i) MSCs identified at the tissue level, where spindle-shaped cells positive for CD44 and CD90 were in the perivascular niche; and ii) the establishment of an *ex-vivo* cell model using enzymatic digestion of AAA tissue, where cells expressed mesenchymal markers (CD44, CD73, CD90, and CD105) and lacked endothelial or hematopoietic markers (CD34, CD14, and CD45). We also identified stemness gene expression (OCT-4, NANOG, and SOX-2) and demonstrated multilineage differentiation, particularly toward osteogenesis. These findings, along with altered immunomodulatory potential, highlight the functional transformation of vascular MSCs under pathological conditions.^4^

The authors assessed MSC presence using single-cell RNA sequencing and immunohistochemical staining, finding that MSC markers were not expressed. Their single-cell RNA sequencing identified 17 clusters, primarily consisting of 10 main cell types, with CD90-CD73-CD105-positive cells in adventitial vasa vasorum. As they suggest, the lack of MSCs may be due to the end-stage nature of AAAs,^1^ where factors such as vascular remodeling, inflammatory infiltrate, systemic conditions, and patient age significantly influence MSC presence. We agree that identifying MSCs with all canonical markers in these conditions is rare, and it is more likely to find a heterogeneous population at various differentiation stages. The clinical data, surgical timing, sample collection, and laboratory practices also influence MSC isolation reproducibility, as all twelve AAA specimens in our study underwent the same digestion and isolation procedures.

We also appreciate the authors' insight into the potential discrepancy due to the different atherosclerotic lesion patterns across arterial regions. This underscores the need for more refined methods to isolate and study MSCs within AAAs. Our team has extensive experience harvesting MSCs from various vascular segments, both fresh and cryopreserved.^5^ We have accounted for the high cellular heterogeneity driven by the atherosclerotic microenvironment. Advanced techniques like single-cell RNA sequencing can provide more sophisticated identification of MSCs, uncovering novel markers beyond the canonical ones.

Further research, including collaborative efforts, is essential to elucidate the role of resident MSCs in AAA disease and to guide the development of more personalized and effective therapies.

## CRediT authorship contribution statement

**Carmen Ciavarella:** Conceptualization, Writing – original draft, Writing – review & editing. **Francesco Alviano:** Conceptualization, Writing – original draft, Writing – review & editing. **Gianandrea Pasquinelli:** Conceptualization, Supervision, Validation, Writing – review & editing.

## Conflict of interests

The authors declared no conflict of interests.
